# Leaf epidermis images for robust identification of plants

**DOI:** 10.1038/srep25994

**Published:** 2016-05-24

**Authors:** Núbia Rosa da Silva, Marcos William da Silva Oliveira, Humberto Antunes de Almeida Filho, Luiz Felipe Souza Pinheiro, Davi Rodrigo Rossatto, Rosana Marta Kolb, Odemir Martinez Bruno

**Affiliations:** 1 Institute of Mathematics and Computer Science, University of São Paulo, USP, Avenida Trabalhador são-carlense, 400, 13566-590 São Carlos, São Paulo, Brazil; 2 Scientific Computing Group, São Carlos Institute of Physics, University of São Paulo, PO Box 369, 13560-970, São Carlos, SP, Brazil; 3 Department of Biological Sciences, Faculty of Sciences and Languages, Univ Estadual Paulista, UNESP. Av. Dom Antônio, 2100, 19806-900, Assis, São Paulo, Brazil; 4 Department of Applied Biology, Faculty of Agriculture and Veterinary Sciences, Univ Estadual Paulista, UNESP, Via de Acesso Prof. Paulo Donatto Castellane S/N. 14884-900, Jaboticabal, São Paulo, Brazil

## Abstract

This paper proposes a methodology for plant analysis and identification based on extracting texture features from microscopic images of leaf epidermis. All the experiments were carried out using 32 plant species with 309 epidermal samples captured by an optical microscope coupled to a digital camera. The results of the computational methods using texture features were compared to the conventional approach, where quantitative measurements of stomatal traits (density, length and width) were manually obtained. Epidermis image classification using texture has achieved a success rate of over 96%, while success rate was around 60% for quantitative measurements taken manually. Furthermore, we verified the robustness of our method accounting for natural phenotypic plasticity of stomata, analysing samples from the same species grown in different environments. Texture methods were robust even when considering phenotypic plasticity of stomatal traits with a decrease of 20% in the success rate, as quantitative measurements proved to be fully sensitive with a decrease of 77%. Results from the comparison between the computational approach and the conventional quantitative measurements lead us to discover how computational systems are advantageous and promising in terms of solving problems related to Botany, such as species identification.

Green plants (**Viridiplantae**) are among the most important living beings in the natural world. They are multicellular photosynthetic eukaryotic organisms forming a clade that includes flowering plants, conifers and other gymnosperms, ferns, clubmosses, hornworts, liverworts, mosses and green algae^1^, providing most of the world’s photosynthetically fixed carbon^2^, and are the basis of all life on earth. According to O.W. Archibold^3^, 11 major types can be recognized forming the majority of earth ecosystems: tropical forests, tropical savannas, arid regions (deserts), Mediterranean ecosystems, temperate forest ecosystems, temperate grasslands, coniferous forests, tundra (both polar and high mountain), terrestrial wetlands, freshwater ecosystems and coastal/marine systems. In South America, forests and savannas predominate^4^, and in the Brazilian territory, the Cerrado is included as one of the most important tropical savannas^5^. Its geographical reach accounts for 22% of the country’s land area (extending marginally into Paraguay and Bolivia), and is the second most important ecosystem in South America, after the Amazon Forest. The World Wide Fund for Nature called it the biologically richest savanna in the world, with about 12,000 plant species, 44% of which are endemic, according to^6^,^7^. Additionally, the Cerrado has been considered a hot-spot, suffering from severe deforestation and agricultural use^8^.

Due to its incredible plant diversity, which is reflected in a great diversity of morphological structures^9^,^10^, the Cerrado ecosystem presents itself as a great opportunity to test methods and approaches to provide new tools for plant identification. To date, the main approach used to identify any plant species is by using morphological traits^11^. This approach is widely based on morphological traits of reproductive organs that are not always found in the plant, such as flowers and fruits^12^. Alternatively, in recent years, some computational approaches have been proposed to identify species based on images of leaves that are often available throughout the year in tropical and subtropical regions of the world^13^,^14^,^15^,^16^,^17^,^18^. Such methods are able to differentiate the species based on leaf image properties, where texture is the main analyzed feature.

Generally, texture is associated to the feel of different materials to human touch. Texture image analysis is based on visual interpretation of this feeling^19^. By this fact, this descriptor indicates smoothness, coarseness and regularity in images^20^. In computational analysis of plant images, assessing texture of leaf surface is related to different characteristics of the plant, e.g. presence and type of trichomas, stomata types, etc., producing different patterns that can be identified. The application of such methods has been used in leaf cross-sections (analyzing internal structures) or on the leaf surface (where subsamples of the entire scanned leaf were analyzed)^13^,^14^,^15^,^16^,^17^,^18^,^21^.

Another possibility to be explored in computational methods is analysing the leaf epidermal surface by its dissociation^22^. The dissociation process is normally used to infer structural patterns such as size, position and density of stomata, as well the distribution and shape of epidermal cells and characteristics of the cuticle, such as striation patterns^22^,^23^. These traits provide important information for plant identification and can even provide important taxonomic characteristics for phylogeny assembly^24^,^25^,^26^,^27^. When analysing the epidermis surface, the anatomical procedures are relatively simple compared to the preparation of transversal cuts^28^, as the process to obtain the leaf surface is done in less than 12 hours, and many samples can be processed at the same time^29^. Furthermore, the full leaf is not needed to identify the plant. Nevertheless, few studies have assessed the morphometry, anatomical structure and texture of leaf epidermis images with identification purposes. In^30^, dissociated samples of leaf epidermis were used, however only one species was analyzed.

The aim of this paper is to present an innovative use of microscopic images of leaf epidermis: by extracting texture features and classifying the species by analysing these characteristics. Computational methods are advantageous because they can provide many useful features to identify species^31^. The feature extraction process was carried out using three different methods: Fourier descriptors, corrosion-inspired texture analysis and local binary patterns and the classification was done using two classifiers: *k*-Nearest Neighbors and linear discriminant analysis.

In order to show the importance of using computational methods to help identify plant species, the same species used in the previous experiments were classified using morphological characteristics. They were obtained manually by quantitative measurements using a light photomicroscope and the AxioVision microscope software from Zeiss. The analysed traits were the stomata density, the guard cell length and the stomatal complex width. A third set of experiments was conducted in order to verify if the texture based approaches and the conventional method using quantitative measurements are invariant to plasticity. Phenotypic plasticity is the morphological and physiological response of individuals to changes in the environment^32^. Factors such as climate change, land use change, invasiveness and resource limitation may cause morphological intraspecies differences, playing an important role when identifying species. To verify the robustness of plant identification methods to handle the variability of plasticity, we also analyzed the texture features of leaves from the same species growing in distinct environmental conditions.

## Materials and Methods

### Epidermal images acquirement system

Leaves were collected from representative woody species of the Cerrado (Table 1) in the IBGE Ecological Reserve (15°56′42.04′′*S*, 47°52′43.74′′*W*) and in the Águas Emendadas Ecological Reserve (15°33′56.84′′*S*, 47°36′04.22′′*W*), both in Brasília, Federal District, Brazil. Authorities from both ecological reserves issued permits to conduct scientific research on the samples. The abaxial surfaces of the epidermis were obtained from three or four leaves (one per individual) of each species. From each collected leaf, a sample of approximately 1 *cm*^2^ was removed from the leaf’s middle region, between the margin and midrib. Dissociation of the leaf epidermis was performed using a 1:1 solution of glacial acetic acid and hydrogen peroxide at 60 °C for 12 hours, or the time required to completely decouple the epidermis (modified from^29^). After this procedure, the abaxial surface of the epidermis was washed in distilled water, stained with safranin and mounted in glycerin. The images were captured at 20× objective lenses, using a *Zeiss Axio Scope A1* optical microscope, coupled to a digital camera (Zeiss AxioCam MRc model). Three images per leaf were used for the classification procedure. For the experiments using manual measurements, the stomata density, as well the guard cell length and the stomatal complex width, were obtained by using the AxioVision software from Zeiss. Stomatal counts were made in three fields (defined at random) per individual sampled at 10× objective lens. The size of the stomata was measured in at least three different fields, randomly set, totaling 90 stomata. In total, 309 microscopic images of epidermal leaf surfaces were obtained from 32 woody plants that are commonly found on our studied vegetation sites (Table 1).

### Feature extraction for classification

Image texture can be defined as a function of the spatial variation in pixel intensities (gray level values). These textural properties can be extracted from the image and mapped on the form of a feature vector that will represent this image in a process called feature extraction. Afterwards, the texture patterns of each image need to be recognized to identify which texture pattern they belong to, which leads to a problem of texture classification. For this purpose, the texture patterns of all images under analysis are submitted to a classifier, which predicts the classes in each image considering *T*_1_ images for training and *T*_2_ images for testing.

Previous to feature extraction, some procedures can be used to enhance the image. For example, external interference could lead to obtaining dark, noisy or bright images, preventing capturing the original characteristics. Therefore, these procedures minimize the changes caused by these external interferences. The epidermis images obtained for this experiment underwent a staining procedure, which led to obtaining images with the original colour change of the leaves. For this reason, we converted the images to grayscale and then adopted a procedure for contrast enhancement to ensure there was no interference of the color samples in the feature extraction process. This procedure is described later on.

Three types of feature descriptors were used to describe each epidermis image, which are Fourier^33^,^34^,^35^ with two approaches for feature representation: circular angular and circular, Corrosion-Inspired Texture Analysis^36^ and Local Binary Pattern^37^. All these methods are described immediately after the description of the enhancement procedure. We carried out the classification using *k*-Nearest Neighbors and linear discriminant analysis classifiers.

#### Image Enhancement

In this procedure, histogram stretching was used to increase the image contrast. Let an image *f*(*x*, *y*), the enhanced image *g*(*x*, *y*) is obtained by


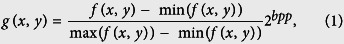


where *bpp* is the number of bits per pixel of the image *f*(*x*, *y*). In our application, the image has 256 gray levels and *bpp* = 8. In the enhanced image *g*(*x*, *y*), 1% of data is saturated at low and high intensities of the original image.

#### Fourier Descriptors

Fourier descriptors, proposed originally by Cosgriff in 1960^33^, are a representation of a periodic signal through the coefficients of a finite combination of complex sinusoidal ordered by their frequencies. Coefficients of the sum of sines and cosines are obtained from the discrete Fourier transform (DFT). Since the image is at the frequency domain, complex values can be used as feature descriptors to represent an object or texture^34^,^35^. Lower frequency coefficients are shifted to extremities of the spectrum. Thus, a shift operation needs to be carried out, which moves the origin of the Fourier transform to the central coordinates at the frequency domain. Low frequency components describe the most relevant information of the behavior of a signal. On the other hand, high frequency components are related to abrupt changes and noise.

### Fourier Circular-Angular

After the shift operation, *F*(*u*, *v*) is partitioned into 64 sectors using eight circular rings and eight angles equally spaced over the image. Circular rings were calculated using radii equal to 3, 6, 9, 12, 15, 18, 21 and 24 pixels of distance. A total of 64 descriptors were given by the sum of the absolute values of each sector of the spectrum.

### Fourier Circular

*G* circular rings using radii equal to 1, 2, …, 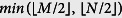, where the image has *M* × *N* pixel size, are calculated over *F*(*u*, *v*) after the shift operation. For each circular ring, the sum of all the absolute values of the spectrum from the origin to the circular ring is performed obtaining G descriptors for each image. As the images obtained in this experiment have sizes of 2080 × 1540 pixels, 769 descriptors are generated per image.

#### Corrosion-Inspired Texture Analysis

Corrosion-Inspired Texture Analysis (CITA)^36^ is a feature descriptor based on concepts from the process of metal pitting corrosion modeling and cellular automata. In this approach, a texture image is considered as a metal surface and thus represents the initial state of the cellular automaton. Each pixel in the image is considered as a cell of the automaton and, therefore 256 initial states are considered, which correspond to 256 gray levels of the image. The gray levels represent the depth of corrosion in a given surface, where 0 means no corrosion, and 255 means the greatest depth of corrosion. An update rule based on the analysis of the central pixel/cell and its neighborhood is set to update the state of each cell at time *t* + 1.

Let an image be considered as a grid *I* of size *N* × *M*. Each cell (*i*, *j*), where *i* ∈ [1, *N*] and *j* ∈ [1, *M*], is updated at time *t* + 1 according to the level of depth calculated from the difference between the state of the central cell and the lowest state value within its neighborhood at time *t*.


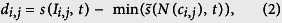


where *s*(*I*_*i,j*_, *t*) is the state of the central cell *I*_*i,j*_ and 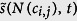 is the set of states of the cells in the neighborhood of *I*_*i*,*j*_.

Thus, the state of the cell *I*_*i*,*j*_ at time *t* + 1 is updated according to:


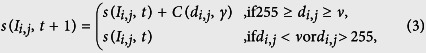


where *v* is a surface roughness parameter, *C* is the level of corrosion to be applied, considering *d*_*i*,*j*_ and the pitting power parameter *γ* ∈ [0, 1]. *γ* is the resistance of the metal to corrosion in a certain environmental condition.


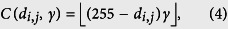


where 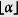 is the floor of *α*.

This process is performed *T* times. For each iteration, the cumulative mass of corroded metal of the CA-based model is used to construct the texture descriptor of size *T* for each image. In this context, 200 iterations were used to classify the image epidermis and the parameters were set up as *v* = 2 and *γ* = 0.05. With these parameters, 200 CITA descriptors were generated.

#### Local Binary Pattern

Local Binary Pattern (LBP)^37^ is an operator of grayscale texture that characterizes the spatial structure of local binary patterns in the image in a circular neighborhood at any spatial resolution. This approach suggests the image consists of micro-patterns. Given a central pixel in the image, a number of patterns is computed by comparing its value with the pixels of its neighborhood:


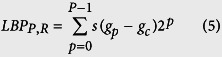



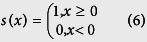


where *g*_*c*_ is the gray level of the central pixel, *g*_*p*_ the gray level of the neighbor *p*, *P* is the number of neighbors and *R* is the radius of the neighborhood. Let an image of size *N* × *M* pixels, the LBP pattern of each pixel (*i*, *j*), 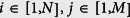, is determined and a histogram of these patterns is calculated to represent the texture image. Consequently, a feature vector with 256 positions (the same number of gray levels) is generated for each image.

## Experiments and Results

### Texture analysis vs traditional methods

**Figure 1:**
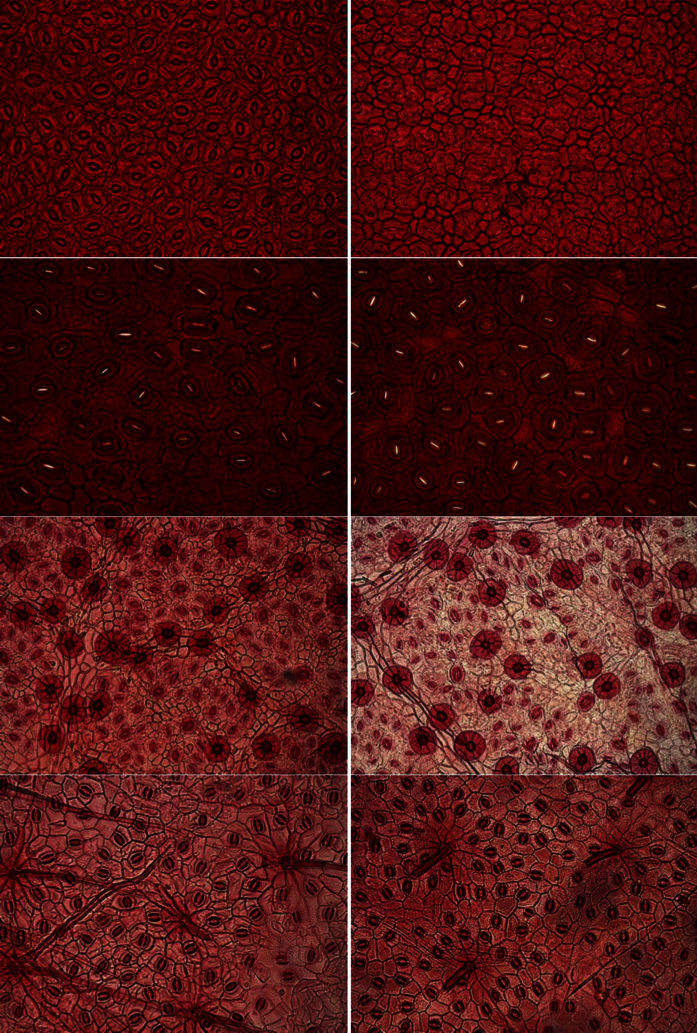
Samples of epidermal images with low variation in images of the same species (row). From top to bottom: *Ilex affinis*, *Myrsine guianensis*, *Handroanthus impetiginosus* and *Xylopia sericea*.

**Figure 2:**
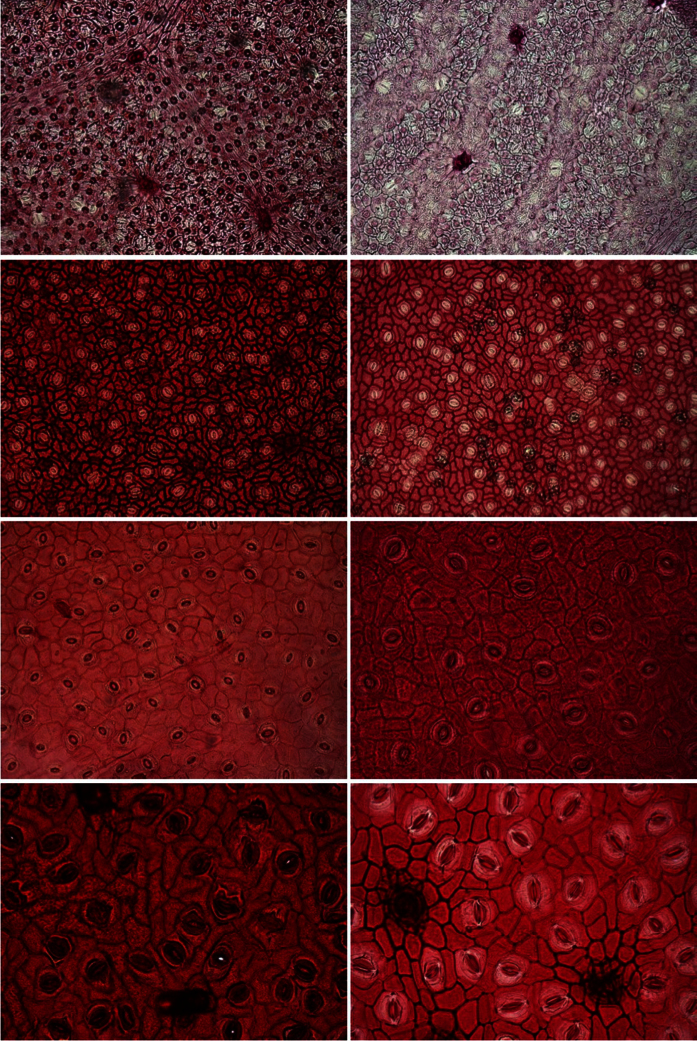
Samples of epidermal images with wide variations in images of the same species (row). From top to bottom: *Miconia cuspidata*, *Tapirira guianensis*, *Symplocos mosenii* and *Guapira noxia*.

Following the procedure described in the Materials and Methods section, 300 samples of size 2080 × 1540 pixels distributed over 32 distinct woody species (see Table 1 for details) were obtained. Figures 1 and 2 show two samples of epidermis images of the same species per row. In the first figure, images with intra species similarity are shown, i.e., there is no significant variation of the images obtained from one individual to another. However, this is not the case for all species, as shown in Fig. 2, where wide variations in images of the same species (when considering different individuals) can be seen. This context makes the classification problem even more challenging.

Three texture descriptors were used to describe the images and subsequently perform the classification, which were Fourier, CITA and LBP descriptors. For the Fourier descriptors two approaches were used. The first one used only the Fourier circular descriptors, while the second one used the concatenated vectors of Fourier circular and Fourier circular-angular. The number of descriptors obtained varied from 200 to 833, therefore the Principal Component Analysis (PCA), a technique to reduce the dimensionality, was used for all the feature descriptors so that a smaller number of features could be used. PCA is an orthogonal linear transformation that converts a number of possibly correlated variables into a set of values of uncorrelated variables called principal components^38^. The first principal component has the greatest variance in the data, the second principal component and succeeding components have the highest variability in descending order.

A supervised classification was performed to assign each sample to a plant species. This process is called supervised because the species of the training set are known and the aim is to use a function to classify a new observation in one of the given species. A validation scheme termed stratified 6-fold cross-validation^39^ was used. The set of samples was equally divided into six mutually exclusive subsets and samples of five subsets were used for training, and samples of one set were used for testing. This procedure was performed 6 times alternating the testing subset so that all samples were classified. In the end, the success rate for the data set was calculated. This procedure was executed 10 times to obtain the standard deviation.

To classify the samples, two classifiers were used: *k*-Nearest Neighbor (*k*-NN)^40^,^41^, with *k* = 1 (experiments with *k* = 1, 3, 5 and 7 were carried out. For the analysed data, *k* = 1 obtained the best performance. *k*-NN for *k* = 1 is also called nearest neighbor algorithm), and Linear Discriminant Analysis (LDA)^42^. *k*-NN associates a class to a sample according to the most repeated class of the surrounding neighborhood. LDA maximizes the ratio of between-class variance and within-class variance achieving the maximal separability. These procedures for classification were applied to the set of epidermal images and high probabilities of correct classification were obtained performing 96.6% of success rate using Fourier descriptors. All the results were compared in Tables 2 and 3 for *k*-NN and LDA, respectively.

Moving on further, the proposed method was compared to the traditional approach, where quantitative measurements were obtained manually from the epidermis images. The following features were considered: stomata density, the guard cell length and the stomatal complex width. The same 32 species and 300 samples used for the computational experiments were also used for the morphological experiment, as well the stratified 6-fold cross-validation scheme. The results using quantitative measurements are 61.33% with *k*-NN and 58.47% with LDA. For each of the texture methods, the three descriptors obtained manually (density, length and width) were added to the texture feature vector to verify the impact of using both features together for plant species recognition. It can be observed in Tables 2 and 3 that morphological characteristics may increase up to 10% success rate when combining both features.

### The effect of plasticity on identification

Plasticity is an important characteristic that makes it difficult to conventionally identify plant species because the individuals from the same species, which grow in different vegetation formations with different environmental factors, can present different morphological features^43^. The database used for the experiments contains 300 images from 32 species, including six samples of *Tapirira guianensis* species grown in a gallery forest environment. The results presented in Tables 2 and 3 at column ‘% *T. g*.’ show the identification success rate of the *Tapirira guianensis* species grown in a gallery forest environment.

To verify if the approaches used in this work (the computational method and the traditional method) are invariant to plasticity, nine images of *Tapirira guianensis* species grown in a marsh camp environment were added to the database making a total of 309 images. For this new dataset, two approaches were considered. Firstly, images of *Tapirira guianensis* grown in a gallery forest and in marsh camp environment were placed together in the database and classified using a stratified 6-fold cross-validation scheme (Column ‘% *T. g. joint*’ in Tables 2 and 3). Secondly, the nine samples of *Tapirira guianensis* species grown in a marsh camp environment were considered as the test set and the 300 images of the database were considered as the training set (Column ‘% *T. g. split*’ in Tables 2 and 3). The first approach analyses the increase in the morphological variability, when samples of the same species grown in another environment are added. The second one analyses the direct effect of plasticity on the identification methodology. In this case, for successful identification, methods must be able to observe characters that are less biased for the growing environment. Considering only samples of *Tapirira guianensis* from the gallery forest, the recognition rate was 100% using the traditional method. However, when adding samples obtained from *Tapirira guianensis* grown in a marsh camp, which increases the variability due to the plasticity (‘309 images’, column ‘% *T. g. joint*’, Tables 2 and 3), the success rate using the traditional methodology fell sharply, while the texture descriptors remained robust, maintaining the high success rate of the *Tapirira guianensis* species identification.

Considering the experiment where the samples from the two grown environments were split, training with samples grown in a gallery forest and testing using samples grown in a marsh camp (‘309 images’, column ‘% *T. g. joint*’, Tables 2 and 3), the results of the traditional approach showed a high decrease. It changed from 100% to 44% of success rate assessed by the *k*-NN classifier and to 0% estimated by the LDA classifier. On the other hand, in the case of the *k*-NN classifier, the texture descriptors had a high decrease, achieving a success rate less than 50%. Taking into account the LDA classifier, the results were better, showing 67% of success rate for most of the texture descriptors. The exception of this decrease was the LBP method, which had a high success rate for both classifiers, achieving 78% for *k*-NN and 89% when using the LDA, and 100% of success when combined with the traditional method using the LDA.

## Discussion

Analysing morphological and anatomical traits has helped to discriminate species for a long time^23^,^43^. In this context, information about leaf epidermis features (distribution pattern of epidermal cells, types of trichomes and stomata, shape of guard cells and cuticular ornamentation) have been quite useful for taxonomical approaches^44^,^45^,^46^. However, these features could be limited to discriminating a large number of species. Considering this, computational analysis of the epidermis texture provides many suitable descriptors for plant discrimination, which shows it is a promising approach in this task.

In our study, four different approaches were used to obtain information about leaf epidermis to classify species, two of which were related to feature extraction based on Fourier transform. Both of these showed the best results. Furthermore, CITA and LBP feature descriptors were used, also proving to be effective to identify plant species. All the tests were carried out using both *k*-NN and LDA classifiers and similar results were obtained in most of the cases, showing the consistency of the results. Considering the plasticity experiment, columns ‘% *T. g. joint*’ and ‘% *T. g. split*’ in Tables 2 and 3, it can be observed that the LDA had a much better result than *k*-NN. The *k*-NN is a very simple classification method that proved to be very useful in most of the applications, while LDA is more sophisticated and uses linear transformations to explain the data better. Therefore, when the morphological variability between the training and testing sets are high, the result is also dependent on the adopted classifier, and the LDA is a better option.

The ability of the Fourier descriptors to concentrate the low frequency components separately from the high frequency components enables us to analyse epidermal tissue (regular cells with coating function) separately from specialized cells (stomata) and epidermal appendices (trichomes). Thus, attributes of low and high frequency can be solely compared between samples and these comparisons are joined to enhance the separation of different species and decrease the distance between samples of the same species. Therefore, Fourier descriptors showed the best classification rate, achieving a very impressive success rate higher than 96%. CITA and LBP descriptors have similar methodologies for texture analysis considering the behavior of central pixel neighborhood for feature extraction. CITA considers regions with similar values as belonging to the same local surface and tries to maintain these regions throughout the iterations. These regions may be the epidermis tissue and regions within the structure of stomata. Furthermore, CITA attempts to erode regions with high abrupt differences so that structures on the epidermis can be analysed. Thus, CITA achieves a reasonable result in plant species classification. LBP accounts for patterns of neighbors by simply analyzing if they have a value higher or lower than the central pixel without considering the difference ratio. When considering the plasticity and a large variability from the training and testing sets, the LBP method obtained the best result. Among the texture descriptors evaluated, Fourier and LBP descriptors are the best options. The choice between them has to be made according to the variability of the samples used in the experiment.

Concerning the comparison between computational methodologies based on texture and the conventional approach using manual measurements, significant differences were observed. The methods based on texture features were better able to classify the species than just the quantitative data, which are more laborious to obtain. Texture methods are able to identify plant species even when the species presents plasticity in their quantitative measurements from the epidermis structures. These quantitative values can change according to the environment, however the texture information remains concise. An analysis using manual measurements considers only the shape and density of the stomata, whereas the texture analysis considers spatial orientation and geometric arrangement of the stomata, as well as their quantitative characteristics. The orientation of the stomata is an important attribute as in certain groups of species, it can be used as an important discriminating attribute, as in the case of monocotyledons and pine species. Furthermore, as the identification method based on texture uses the entire image of the epidermis, stomata are not segmented, thus epidermal tissue patterns and also other structures that might occur in some species, such as trichomes are also considered. The combination of all these features allows for a rich analysis, able to provide a strong and unique identity to different plant species.

The texture information of the epidermis when combined with the manual measurements of the stomata (density, length and width) enhance the information concerning species identification. When texture and morphological features are used together, the success rate may increase up to 10%. The main advantage of texture methods is that it is no longer necessary to segment the stomata. The stomata segmentation process is a difficult task, as the color and contrast of the stomata varies for each species and, in many cases, an automatic segmentation methodology is not feasible. Considering that the texture method is automatic and can be analysed more quickly without manual intervention of the images while maintaining a good success rate, depending on the experiment, it can be used without combining it with the quantitative measurements.

## Conclusions

The main method used to identify any plant species is based on the morphological analysis. Nevertheless, identifying plant species can become a difficult task for botanists and other researchers not specialized in plant taxonomy. In this context, computational approaches have became an important tool to help in the task of identifying plant species. Considering the efficiency of the texture descriptors to discriminate plant species by epidermis images and the simplicity of this approach, an innovative concept is proposed that can investigate common texture features extracted from the taxonomic hierarchical levels, which could help taxonomists to classify new unknown plant species using a pattern recognition system. These new discrimination strategies associated with extracting features of images of leaves can open up new possibilities for classification methods of modern taxonomy.

## Additional Information

**How to cite this article**: Silva, N.R. *et al*. Leaf epidermis images for robust identification of plants. *Sci. Rep*. **6**, 25994; doi: 10.1038/srep25994 (2016).

## Figures and Tables

**Table 1:** List of species from which we obtained the leaf epidermis images.

Species (Family)
*Baccharis linearifolia* (Lam.) Pers. (Asteraceae)
*Byrsonima laxiflora* Griseb. (Malpighiaceae)
*Calophyllum brasiliense* Cambess. (Clusiaceae)
*Campomanesia velutina* (Cambess.) O. Berg (Myrtaceae)
*Clusia criuva* Cambess. (Clusiaceae)
*Copaifera langsdorffii* Desf. (Fabaceae)
*Cupania vernalis* Cambess. (Sapindaceae)
*Duguetia furfuracea* (A.St.-Hill.) Saff. (Annonaceae)
*Eriotheca candolleana* (K. Schum.) A. Robyns (Malvaceae)
*Esenbeckia pumila* Pohl. (Rutaceae)
*Gaylussacia brasiliensis* (Spreng.) Meisn. (Ericaceae)
*Guapira noxia* (Netto) Lundell (Nyctaginaceae)
*Hymenaea stigonocarpa* Mart. ex Hayne (Fabaceae)
*Ilex affinis* Gardner (Aquifoliaceae)
*Matayba guianensis* Aubl. (Sapindaceae)
*Maytenus floribunda* Reissek (Celastraceae)
*Miconia cuspidata* Naudin (Melastomataceae)
*Miconia chamissois* Naudin (Melastomataceae)
*Myrsine guianensis* (Aubl.) Kuntze (Myrsinaceae)
*Myrsine ferruginea* (Ruiz & Pav.) Spreng. (Myrsinaceae)
*Ouratea hexasperma* (A.St.-Hill.) Baill. (Ochnaceae)
*Plenckia populnea* Reissek (Celastraceae)
*Pseudobombax longiflorum* (Mart. & Zucc.) A.Robyns (Malvaceae)
*Roupala montana* Aubl. (Proteaceae)
*Rourea induta* Planch. (Connaraceae)
*Salacia crassifolia* (Mart. ex Schult.) G. Don (Celastraceae)
*Symplocos mosenii* Brand (Symplocaceae)
*Symplocos nitens* (Pohl) Benth. (Symplocaceae)
*Handroanthus impetiginosus* (Mart. ex DC.) Mattos (Bignoniaceae)
*Tapirira guianensis* Aubl. (Anacardiaceae)
*Virola sebifera* Aubl. (Myristicaceae)
*Xylopia sericea* A.St.-Hil. (Annonaceae)

**Table 2:** Classification accuracy of 32 plant species.

Feature	*k*-NN						
	300 images			309 images			
	*#*	% (±std)	% *T. g.*	*#*	% (±std)	% *T. g. joint*	% *T. g. split*
Fourier Circular + Circular-Angular	20	96.00 (±0.05)	100	20	94.17 (±0.06)	80	33
Fourier Circular + Circular-Angular + Quantitative	23	98.67 (±0.03)	100	23	96.76 (±0.05)	80	44
Fourier Circular	19	95.00 (±0.06)	83	19	93.20 (±0.06)	67	33
Fourier Circular + Quantitative	22	97.33 (±0.04)	100	22	95.79 (±0.05)	80	44
CITA	19	74.33 (±0.12)	100	21	74.11 (±0.12)	80	44
CITA + Quantitative	22	84.67 (±0.09)	100	24	84.14 (±0.09)	80	44
LBP	29	70.00 (±0.13)	67	33	72.49 (±0.13)	80	78
LBP + Quantitative	32	80.67 (±0.11)	100	36	81.88 (±0.10)	87	56
Quantitative (Density + Length + Width)	3	61.33 (±0.15)	100	3	57.61 (±0.15)	33	44

The results are described by the number of PCA components (*#*), success rate and standard deviation (std) using Fourier, CITA and LBP feature descriptors and *k*-NN as classifiers. Moreover, the results are presented in two modes. The first one shows the success rate for 300 images, in which images of *Tapirira guianensis* only from the gallery forest are used. The second one, labeled as ‘309 images’, shows the results including nine images of *Tapirira guianensis* from a marsh camp to draw a comparison of the classification rate with samples of the same species which grew in different environments. The success rate of identifying the *Tapirira guianensis* species using stratified 6-fold cross validation is shown in the columns ‘% *T. g*.’ and ‘% *T. g. joint*’, respectively, for 300 and 309 images and the column ‘% *T. g. split*’ shows the result considering the nine images of *Tapirira guianensis* from the marsh camp as the testing set and the remaining 300 images as the training set.

**Table 3:** Classification accuracy of 32 plant species.

Feature	LDA						
	300 images			309 images			
	*#*	% (±std)	% *T. g.*	*#*	% (±std)	% *T. g. joint*	% *T. g. split*
Fourier Circular + Circular-Angular	45	96.60 (±1.27)	100	46	94.92 (±0.63)	89	67
Fourier Circular + Circular-Angular + Quantitative	48	97.43 (±0.39)	100	49	96.28 (±0.53)	89	67
Fourier Circular	31	95.83 (±0.63)	100	40	94.63 (±0.79)	92	67
Fourier Circular + Quantitative	34	97.43 (±0.49)	100	43	96.34 (±0.82)	92	67
CITA	64	78.13 (±1.35)	83	55	78.93 (±1.82)	86	56
CITA + Quantitative	67	86.87 (±1.31)	83	58	85.28 (±2.38)	86	44
LBP	87	82.83 (±1.39)	70	80	83.43 (±1.23)	83	89
LBP + Quantitative	90	91.10 (±1.44)	70	83	88.71 (±1.90)	83	100
Quantitative (Density + Length + Width)	3	58.47 (±1.02)	100	3	55.28 (±0.62)	36	0

The results are described by the number of PCA components (*#*), success rate and standard deviation (std) using Fourier, CITA and LBP feature descriptors and the LDA as classifiers. Moreover, the results are presented in two modes. The first one shows the success rate for 300 images, in which images of *Tapirira guianensis* only from the gallery forest are used. The second one, labeled as ‘309 images’, shows the results including nine images of *Tapirira guianensis* from the marsh camp to draw a comparison of the classification rate with samples of the same species which grew in different environments. The success rate of identifying the *Tapirira guianensis* species using stratified 6-fold cross validation is shown in columns ‘% *T. g*.’ and ‘% *T. g. joint*’, respectively, for 300 and 309 images and the column ‘% *T. g. split*’ shows the result considering the nine images of *Tapirira guianensis* from the marsh camp as the testing set and the remaining 300 images as the training set.
